# The impact of the oral cavity in febrile neutropenia and infectious complications in patients treated with myelosuppressive chemotherapy

**DOI:** 10.1007/s00520-019-04925-8

**Published:** 2019-06-20

**Authors:** Judith A. E. M. Zecha, Judith E. Raber-Durlacher, Alexa M. G. A. Laheij, Anneke M. Westermann, Joel B. Epstein, Jan de Lange, Ludi E. Smeele

**Affiliations:** 1 Department of Oral and Maxillofacial Surgery, Amsterdam UMC, University of Amsterdam, Meibergdreef 9, 1105 AZ Amsterdam, The Netherlands; 2 Department of Oral Medicine, Academic Centre for Dentistry Amsterdam, University of Amsterdam and VU University, Amsterdam, The Netherlands; 3 Department of Oncology, Amsterdam UMC, University of Amsterdam, Amsterdam, The Netherlands; 4 Cedars-Sinai Health System, Los Angeles and City of Hope Cancer Center, Duarte, CA USA; 5 Academic Centre for Dentistry, (ACTA), University of Amsterdam, Amsterdam, The Netherlands; 6 Department of Head & Neck Oncology & Surgery, Netherlands Cancer Institute- Antoni van Leeuwenhoek, Amsterdam, The Netherlands

**Keywords:** Febrile neutropenia, Oral infection, Dental health, Oral mucositis, Cancer chemotherapy

## Abstract

Febrile neutropenia (FN) is an inflammatory response causing fever that may develop during cancer therapy-induced neutropenia. FN may herald life-threatening infectious complications and should therefore be considered a medical emergency. Patients presenting with FN are routinely subjected to careful history taking and physical examination including X-rays and microbiological evaluations. Nevertheless, an infection is documented clinically in only 20–30% of cases, whereas a causative microbial pathogen is not identified in over 70% of FN cases. The oral cavity is generally only visually inspected. Although it is recognized that ulcerative oral mucositis may be involved in the development of FN, the contribution of infections of the periodontium, the dentition, and salivary glands may be underestimated. These infections can be easily overlooked, as symptoms and signs of inflammation may be limited or absent during neutropenia. This narrative review is aimed to inform the clinician on the potential role of the oral cavity as a potential source in the development of FN. Areas for future research directed to advancing optimal management strategies are discussed.

## Introduction

Fever is a manifestation of multiple pathways including the release of pro-inflammatory cytokines (including interleukin (IL)-1, IL-6, and tumor necrosis factor (TNF)-alpha) as a consequence of infection or inflammation [1]. Fever that develops during cytotoxic cancer therapy-induced neutropenia, or “febrile neutropenia” (FN), is a medical emergency. FN may be an early and only indication of a serious infection, since profoundly neutropenic patients are unable to mount a robust inflammatory response, and signs and symptoms of inflammation are typically attenuated or absent. In these patients, infections may rapidly progress into life-threatening complications (e.g., systemic inflammatory response syndrome (SIRS), (severe) sepsis, and septic shock). It is critical to early recognize FN (oral temperature > 38.5 °C or two consecutive readings of > 38.0 °C for 2 h and an absolute neutrophil count < 500 μ/L or expected to fall below this threshold), and depending on the estimated risk to develop life-threatening complications, initiate empiric systemic broad-spectrum antibacterial therapy and other supportive care measures promptly [2]. Moreover, FN is associated with higher morbidity, unplanned or prolonged hospitalization, potential intensive care admission, and can necessitate chemotherapy (CT) dose reductions and/or treatment delays, which may lead to poorer clinical outcome and survival [2, 3].

If a patient presents with FN while on myelosuppressive CT, one should assume that this is due to infection, particularly if there is no evidence for an alternative explanation (e.g., transfusion reactions, medication allergies and toxicities, vasculitis or other inflammatory conditions, and tumor(lysis)-related fever) [4]. Nevertheless, patients must be carefully evaluated for potential infection. These patients are routinely subjected to careful history taking, physical examination, chest X-ray, and microbiological evaluations. However, infections can be documented clinically in only 20–30% of febrile episodes, whereas a causative microbial pathogen cannot be identified in the majority (> 70%) of cases [5–10], and the cause of the fever may remain unidentified (fever of unknown origin; FUO).

Neutropenia risk depends on patient-, tumor-, and treatment-related factors. Although CT dose intensity and myelotoxicity represent significant risk factors, neutropenia and its complications may occur after treatment with almost any type of CT [11]. However, not all neutropenic patients develop fever, and not all episodes of FN progress into life-threatening sepsis. CT regimens can be classified as high, intermediate, and low risk for FN [12]. Those with > 20% FN risk are considered high risk. This includes, but is not limited to, patients treated with high-dose CT with or without hematopoietic stem cell transplantation (HSCT). Most CT regimens used for the treatment of adult solid tumors and lymphoma are rated as intermediate (between 10 and 20%) risk for FN, whereas low-risk cytotoxic regimens are typically considered to be associated with FN risk < 10% [13, 14]. Tools aimed to identify patients with FN at low risk of serious complications have been developed [15]. However, identification of high-risk patients remains a challenge [16]. It is well recognized that oral mucositis (OM) as well as gastro-intestinal mucositis may cause fever [17, 18] and predisposes to systemic translocation of microorganisms [19, 20]. If a patient presents with FN, the physician routinely performs an oral inspection to assess whether ulcerative OM or mucosal infections are present. However, fever may be also caused by inflammation and infection at other oral sites, including the periodontium, the dentition, and salivary glands. Most frequently, this involves asymptomatic chronic infections that were present before the initiation of CT. These infections may exacerbate and present with redness, swelling, and pain [21], but they may also be asymptomatic or manifest with only minimal signs of inflammation. Nevertheless, these predominantly asymptomatic infections (particularly those of the periodontium) may contribute to systemic inflammation [22] and risk of bacteremia and are associated with FN [23–25]. Unfortunately, these infections likely remain undiagnosed by simple oral inspection [26]. In order to ascertain diagnosis and management of these “hidden” oral infections, the National Institute of Health [27] stated that all patients should receive a comprehensive oral examination by a dental professional and potential foci of infection should be eliminated prior to the start of CT. In most centers, however, this is may be only routinely performed in patients considered at high risk for complications: i.e., patients scheduled for HSCT and those undergoing (chemo)radiation therapy to the head and neck, whereas patients treated with other myelosuppressive CT regimens are not typically referred for pre-treatment oral evaluation. It is thus conceivable that particularly in these patients, FN may have originated from an oral source that remained undiagnosed.

This narrative review is aimed to inform the oncologist of current evidence of the contribution of oral infections and non-infectious conditions (e.g., OM) to the development of FN and infectious complications associated with myelosuppressive CT. Areas for future research directed to developing optimal management strategies are presented.

## The impact of myelosuppressive CT on the oral ecosystem

Oral homeostasis is maintained due to complex and finely tuned interactions between the host and resident microorganisms. Having cancer, myelosuppressive cancer treatment regimens and concurrent medications, including antibiotics, disturb the equilibrium of the oral ecosystem via direct and indirect mechanisms, and contribute to the development of oral complications and infectious sequelae.

The mouth houses a diverse microbial community, harboring over 700 species of bacteria that colonize the hard surfaces of teeth and soft tissues as biofilms [28]. Residing microbes prevent colonization and overgrowth of opportunistic and pathogenic microorganisms. Studies suggest that treatment with myelosuppressive CT induces alterations of the oral microbiome (Table 1).

**Table 1:** Myelosuppressive CT and its impact on the oral microflora

Author	Results	Patient group
Peterson et al. [29]	Potentially pathogenic bacteria could be identified in granulocytopenic patients, e.g., - Staphylococcus spp. - Gram negative enteric bacilli	Acute leukemia
Bergmann et al. [30]	Enterobacteriaceae, *Enterococcus faecalis,* Candida spp. Identified following CT	Acute leukemia
Napenas et al. [31]	> 60% of identified species were exclusively found post-CT	Breast cancer
Ye et al. [32]	Decreased oral microbial diversity and shifts in the microbiological composition	Pediatric cancer
Galloway-Peña et al. [33]	Decrease of oral and stool microbial α-diversity during remission-induction CT in patients that developed a microbiologically documented infection	Acute myeloid leukemia
Dahlen [34]	Shifts of oral mucosal microflora increases the likelihood of infections with enterococci and staphylococci	Immunocompromised patients receiving cytotoxic CT
Kennedy et al. [35] Costa et al. [36] Soga et al. [37]	Bacteremia with CONS may originate from oral mucosal membranes	HSCT [35] Immunocompromised patients including cancer patients [36] HSCT [37]
Soga et al. [38]	MRSA was detected in the oral cavity following HSCT, especially during OM peak severity	HSCT

*CT* chemotherapy, *CONS* coagulase-negative staphylococci, *HSCT* hematopoietic stem cell transplantation, *MRSA* methicillin-resistant staphylococcus spp., *OM* oral mucositis

As a result of cytotoxic CT and other concurrent medications, patients may develop hyposalivation (reduced salivary flow) [39]. Saliva has a key role in maintaining a stable oral ecosystem as it contains peptides and proteins that have many different qualities. These include antibacterial, antifungal, and antiviral functions as well as lubricant, buffering, remineralization, and digestion properties [40]. A lack of these salivary defense mechanisms may contribute to oral dysbiosis contributing to an increase of the incidence and severity of complications, including OM, oral mucosal infections, and dental caries [41, 42].

## The potential contribution of oral sources of infection and inflammation to FN and infectious complications

The oral cavity is the primary gateway to the human body as it is contiguous with the tonsils, pharynx, esophagus, eustachian tube, middle ear, trachea, lungs, nasal passages, and sinuses. Microorganisms colonizing the oral cavity may migrate via contiguous epithelial surfaces to these anatomical sites [43]. In addition, oral microorganisms and inflammatory products may translocate into the circulation through ulcerated periodontal pockets and breached epithelial lining of mucous membranes and disseminate systemically [44, 45]. This may contribute to fever and infectious complications in neutropenic or otherwise immunocompromised patients [23, 46, 47] and has been reported to contribute to increased risk to early non-tumor-related mortality in HSCT recipients [48–50]**.**

## Oral mucositis

OM is an inflammatory condition of the oral mucosa induced by CT and/or radiation therapy. Most often, it manifests at the buccal and labial surfaces, ventral surface of the tongue, floor of the mouth, and the soft palate [51]. Mucosal changes can range from erythema to extensive ulcerations and hemorrhage. Patients experience OM as a painful condition associated with significant suffering and a negative impact on the quality of life [51, 52]. Ulcerative OM may not only act as a portal of entry for microorganisms and inflammatory products, but as it often compromises food intake, oral medication use, and oral hygiene, it may also contribute indirectly to infection risk [52, 53].

The majority of patients treated with high-dose CT regimens (with or without HSCT) develop OM (all grades), whereas its incidence in less intensive forms of CT is estimated to vary between 0 and 52% [54, 55] (summarized in Table 2).

**Table 2:** The frequency of oral mucositis (all grades) among patients treated with chemotherapy for different types of malignancies

Author	Oral mucositis incidence (%)	Patient group	Chemotherapy regimen
Jensen et al. [56]	22	Breast cancer	Cyclophosphamide, epirubicin or methotrexate, and 5-fluorouracil
Al Ibraheemi et al. [57]	81.3	Breast cancer	Adriamycine, cyclophosphamide, and taxane
Nishimura et al. [58]	76.5 63 57.8 42.9	Breast cancer Colorectal cancer Esophageal cancer Malignant lymphoma	CAF regimen; cyclophosphamide, doxorubin hydrochloride, 5-FU AC; doxorubicin hydrochloride, cyclophosphamide FOLFIRI regimen; irinotecan, leucovorin, bolus 5-FU and a 46-h infusion of 5-FU FOLFOX 4 regimen; oxiliplatin, leucovorin, bolus 5-FU and a 46-h infusion of 5-FU Not described R-CHOP regimen; rituximab, cyclophosphamide, doxorubicin hydrochloride, vincristine sulfate, prednisone (weekly rituximab clinical trial setting).
Laine et al. [59]	43.4	Lymphoma	Hodgkin: ABVD; doxorubicin, bleomycin, velbedacarbazine, or (MOPP)-ABV; mustine, oncocine, procarbazine, prednisone Non-Hodgkin: M-BACOD or M-BECOD; methotrexate, bleomycin, doxorubicin, epiadriamycin, cyclophosphamide, oncovin, dexamethasone
Khan and Wingard. [54]	75 > 66 33 40	Bone marrow transplantation Leukemia Non-Hodgkin lymphoma Solid tumors	Not described

There is a temporal relationship between having ulcerative mucositis, fever, and neutropenia [48, 60–62]. However, the role of neutrophils in the pathogenesis and resolvent of OM has not yet been fully elucidated, and studies on granulocyte stimulating growth factors for the prevention and treatment of OM show conflicting results [25].

Sonis suggested that cells rendered apoptotic or necrotic by CT, release CT-associated molecular patterns (“CRAMPs”), which play an important role in initiating inflammation [63]. When pattern recognition receptors (PRRs) expressed by cells of the innate immune system within the mucosa (e.g., macrophages, neutrophils, and dendritic cells) are exposed to CRAMPS, this results in the release of cytokines that act locally, and also may have distant effects by activating intracellular and intercellular signaling loops [64]. These include the hypothalamic-pituitary-adrenal axis resulting in fever, and effects on the liver resulting in the generation of acute phase proteins. All of which can ultimately result in SIRS and sepsis. Indeed, Blijlevens and colleagues reported that mucositis induces a systemic inflammatory response characterized by fever in neutropenic HSCT recipients, even in the absence of bacteremia [17, 18].

Although OM is not an infectious process, studies suggest that poor oral hygiene and pre-existing oral infections aggravate the severity and duration of toxicity induced mucosal reaction and thereby contribute to the risk to develop FN and infectious complications [53, 65–72] (Table 3).

**Table 3:** Studies reporting a relationship between oral health and oral mucositis, potentially resulting in fever and infectious complications

Author	Results	Patient group
Fernándes et al. [67]	All patients with periodontitis prior to conditioning therapy developed OM. The presence of gingivitis was significantly associated with the frequency of OM	HSCT
Coracain et al. [71]	Patients oral health was predictive of the incidence and severity of OM	HSCT
Kashiwazaki et al. [53] Epstein and Schubert [73]	Treatment of dental pathologies and reducing the oral microbial load resulted in reduction of incidence and severity of OM	HSCT [53] Hematologic malignancies [73]
Kashiwazaki et al. [53]	Maximal CRP levels in peripheral blood and incidence of FN is significantly lower in the group who performed intensive oral hygiene	HSCT
Santos et al. [70]	Treatment of dental pathologies and reducing oral microbial load resulted in shorter duration of OM	HSCT
Khan and Wingard [54]	Presence of oropharyngeal mucositis is an independent risk factor for bacteremia in neutropenic patients	HSCT, non-Hodgkin lymphoma, leukemia
Laine et al. [23, 59]	Association between the presence of oral mucosal ulcers and episodes of fever and sepsis	Lymphoma
Sonis et al. [48] Schuurhuis et al. [62]	OM is associated with higher FN risk as compared with patients without OM	Hematologic malignancies
Ruescher et al. [19] Elting et al. [74] Bochud et al. [75] Marron et al. [76]	OM was most likely origin of bacteremia with oral viridans streptococci, frequently resulting in fever and potentially leading to ARDS and septic shock	HSCT [19] Patients treated with CT [74, 75] Hematologic malignancies [76]
Ebinuma et al. [77]	Maintenance of good oral hygiene may reduce the gene mediating methicillin resistance. The oral cavity may act as a reservoir for harboring this gene	HSCT

*FN* febrile neutropenia, *OM* oral mucositis, *HSCT* hematopoietic stem cell transplantation, *CRP* C-reactive protein, *ARDS* acute respiratory distress syndrome, *CT* chemotherapy

## Oral mucosal infections

In addition to FN associated with OM, oral mucosal ulcerations with a non-mucositis etiology may induce FN. However, from a clinical perspective, it can be difficult to discriminate between such lesions as they may develop simultaneously.

Herpes simplex virus (HSV) is a major pathogen causing mucosal ulceration that may be confused with OM or may aggravate this condition [78]. Most HSV infections occur as a result of reactivation of latent virus in HSV seropositive patients. Upregulation of NF-κB resulting in loss of mucosal barrier in OM may also induce signaling pathways that stimulate virus reactivation [79]. HSV reactivation can be controlled with antiviral prophylaxis or treatment in most patients, although reactivation is still possible despite prophylaxis [54]. Herpetic lesions in patients receiving CT can be painful and may be associated with hemorrhage and necrosis, and bacterial and fungal superinfections. In myelosuppressed patients, HSV infection has atypical clinical manifestations, mainly manifesting as intraoral ulcerations with or without labial involvement. HSV infection may develop at sites, which are usually not affected by OM (e.g., the dorsum of the tongue, gingiva, and the hard palate). Herpetic lesions in compromised hosts may persist for extended periods until recovery from myelosuppression or appropriately treated.

In addition, other herpesviruses, Epstein-Barr Virus (EBV) in particular, may predispose for developing oral mucosal ulcerations during neutropenia and thus may directly or indirectly play a role in FN originating from the oral cavity [80]. Periodontal pockets [81] and salivary glands seem important reservoirs of cytomegalovirus (CMV) [82]. The virus can be shed (via gingival crevicular fluid and salivary glands) in whole saliva forming a main mechanism for transmitting CMV. CMV induces local production of pro-inflammatory cytokines and remains a major cause of morbidity in HSCT recipients [83].

Oral candidiasis is typically caused by opportunistic overgrowth of *Candida albicans*, *C. krusei*, *C. tropicalis*, *C. dubliniensis*, and other commensal oral yeasts. Neutropenia, mucosal damage, depressed cell-mediated immunity, hyposalivation, immunosuppressive medications, and use of antibiotics are risk factors. The most common forms are pseudomembranous and erythematous candidiasis [84]. During oral infection with Candida spp., a large amount of inflammatory cytokines is generated in the oral mucosa, potentially inducing fever [85, 86]. Current prophylactic strategies have reduced systemic candidiasis, although oral and oropharyngeal candidiasis still may have serious consequences. Good oral hygiene is important in addition to antifungal therapy.

Rarely, molds, such as Zygomycetes, Coccidiomycosis, histoplasmosis, and Aspergillus spp., cause oral infections. Typical lesions include ulcers in the oral cavity, tongue, or gingival regions that may progress into invasion of deep tissues, including bone. Early recognition (and confirmation by biopsy), aggressive debridement, and antifungal therapy with extended spectrum azoles are paramount [87].

## Dental pathologies

Pre-existing dental pathologies that may cause FN and infectious complications during myelosuppressive CT include periodontal disease, profound dental caries, and periapical pathology due to infection of the root canal, (partially) impacted teeth, and retained roots [88].

### Periodontal disease

Gingivitis is a localized or generalized inflammation of the gingiva without loss of periodontal attachment, whereas periodontitis is a chronic inflammation of the gingiva, affecting the deeper parts of the periodontium. As dental plaque migrates in apical direction, periodontal attachment degrades, leading to ulcerative periodontal pockets, bone resorption, and tooth loss. About 40–50% of adults has chronic gingivitis, around 35% suffer from moderate periodontitis, whereas the prevalence of severe periodontitis is estimated to range from 10 to 15% in Western populations [26]. Periodontitis risk increases with age (peaks between 50 and 60 years) and is more prevalent in men than in women. The severity of the periodontal disease depends on environmental and host factors (e.g., genetic susceptibility, comorbidities, oral hygiene, and smoking) [89]. Inflamed and infected periodontal tissues can expose up to 35–50 cm^2^ of connective inflamed tissue and may serve as a reservoir of endotoxin (lipopolysaccharide), pro-inflammatory cytokines, and proteolytic enzymes that may spread systemically via ulcerated pocket epithelium and contribute to endothelial dysfunction, a pro-coagulant state, and low-level inflammation [90, 91]. Periodontal infection has been associated with bacteremic seeding of heart valves and prosthetic devices [92], FUO, and a wide range of systemic conditions, including ischemic cardiovascular disease, metabolic syndrome and type 2 diabetes, rheumatoid arthritis, and respiratory diseases, including aspiration pneumonia [93–95].

Table 4 summarizes the literature on gingivitis and periodontitis and their association with bacteremia, FN and infectious complications.

**Table 4:** Presence of periodontal disease and associations with bacteremia, FN, and infectious complications

Author	Results	Patient group
Fernándes et al. [67] Soga et al. 2009 [24] Raber-Durlacher et al. [96]	Presence of periodontitis is associated with the development of bacteremia.	HSCT
Laine et al. [23]	In 58% of the febrile episodes, mild to moderate gingivitis was present In 33% of the febrile episodes, severe gingivitis was present	Lymphoma
Hong et al. [97]	20.3% of patients had severe gingivitis	Pediatric malignancies
Peterson and Overholser [98] Greenberg et al. [65] Overholser et al. [99] Bergmann et al. [46] Laine et al. [23] Meurman et al. [100] Soga et al. [24]	Gingivitis and periodontitis were found to be associated with bacteremia, fever, and sepsis in neutropenic patients	Hematologic malignancies
Russi et al. [64]	FN may be induced by periodontal inflammation and infection	Head and neck cancer

*FN* febrile neutropenia, *HSCT* hematopoietic stem cell transplantation

Ulcerated periodontal pocket epithelium can allow translocation of microorganisms into the bloodstream [101]. Bacteremia may be caused by facultative or strictly anaerobic bacteria likely originating from the periodontium, including Capnocytophaga spp. and *Fusobacterium nucleatum* [102–106]. However, bacteremia with these microorganisms is relatively uncommon, whereas bacteremias with viridans streptococci and CONS occur frequently in profoundly myelosuppressed patients. Increases of gingival viridans streptococci and CONS have been documented during high-dose CT [29], and one study reported that periodontitis may contribute to the risk of bacteremia with viridans streptococci and CONS during the neutropenic phase of HSCT [107]. Periodontal disease may be occult without symptoms and clinical signs that may only be detected upon comprehensive periodontal evaluation and not identified based on general oral examination.

### Miscellaneous odontogenic infections

A number of studies on the potential role of odontogenic infections in the development of FN do not distinguish between periodontal disease and other infections related to the dentition (Table 5).

**Table 5:** Presence of miscellaneous odontogenic infections and associations with bacteremia, FN, and infectious complications

Author	Results	Patient group
Greenberg et al. [65]	Patients who did not receive dental treatment prior to CT: - In 77% of patients, sepsis occurred. - 67% of patients developed acute exacerbations of oral bacterial infections. Patients in which dental infections were treated before CT: - In 25% of the patients, sepsis occurred. - No acute exacerbations of oral bacterial infections occurred.	Acute leukemia
Laine et al. [23]	Patient with febrile episodes had more severe dental pathologies than those without fever (57.6% vs 23.3%)	Lymphoma
Toljanic et al. [108]	Incidence of acute dental problems during febrile episodes of 4%	Solid and hematologic malignancies
Akintoye et al. [109]	23 out of 49 positive blood cultures with microorganisms were likely to originate from the oral cavity; 20 of these bacteremias the periodontium was the most likely origin.	HSCT
Cullen et al. [6]	In 5% of febrile episodes an oral infection was thought to be the focus of infection	Solid tumors and lymphomas
Hong et al. [97]	Weighted prevalence of dental infections of 5.8%	Patients treated with cytotoxic CT
Hong et al. [110]	Weighted prevalence of dental infections was 5.4%; pericoronitis was present in 5.3% of patients	Patients treated with cytotoxic CT
Schuurhuis et al. [62]	Prevalence of 4% of acute exacerbations of chronic oral foci. concluded that asymptomatic chronic oral infections, that had not exacerbated in the previous 3 months, could safely be left untreated without increasing the infectious complications.	Hematologic cancers receiving intensive CT with or without HSCT
Tsuji et al. [111]	Significant higher incidence of systemic infectious and/or inflammatory parameters (temperature > 38.0 C, presence of respiratory symptoms, elevated CRP levels and positive blood cultures) in patients with oral foci of infections, particularly during the first cycle of CT inducing profound myelosuppression.	Hematologic malignancies
Akashi et al. [112]	Development of septic shock in 2 patients of which it was assumed that this was associated with a chronic odontogenic infection. No bacterial evidence was found from the blood cultures.	Hematologic malignancies

*FN* febrile neutropenia, *CT* chemotherapy, *HSCT* hematopoietic stem cell transplantation

## Discussion and recommendations for future research

There is evidence suggesting that infectious and inflammatory processes of the oral cavity may contribute to FN, particularly in patients treated with high-dose CT with or without HSCT. However, most studies are retrospective and include only a small number of patients. Well-powered, prospective observational studies in homogenous groups of patients, including those treated with myelosuppressive CT for solid tumors, are needed to obtain a better understanding of the contribution of OM and oral infections to FN and its potential sequelae.

Particularly, OM [17, 18] and periodontal disease [23, 24, 46, 64, 65, 67, 96, 98–100] seem important in the development of FN, whereas the literature on other potential oral sources of FN (such as mucosal infections, periapical pathologies, and pericoronitis) is scarce. Some studies report infrequent conversion of chronic dental disease to an acute state during CT and conclude that there is no need for the treatment of “asymptomatic” chronic dental infections prior to CT or conditioning therapy for HSCT [62, 108]. However, studies should not focus solely on these acute exacerbations, associated with swelling and pain, since the inflammatory response may be muted or absent due to myelosuppression. It is essential that endpoints of future studies should also include the presence of “asymptomatic” disease, particularly chronic periodontitis which requires comprehensive examination including periodontal probing and appropriate dental imaging.

There is convincing evidence from the periodontal literature that gingivitis as well as periodontitis induces frequent episodes of bacteremia during daily activities such as chewing and tooth brushing [44, 113]. In non-myelosuppressed individuals, these episodes of bacteremia are typically transient. In myelosuppressed and otherwise immunocompromised cancer patients, however, bacteremia risk is likely increased as in addition to breached epithalial barriers, these patients are less capable of clearance of microorganisms from the blood stream, and infectious complications may develop. In addition to hematogenous spread of oral microorganisms and inflammatory mediators, there is evidence that poor oral and dental conditions may predispose for aspiration pneumonia [94, 114].

Systemic dissemination of oral microorganisms should be investigated in more detail in cancer patients treated with myelosuppressive CT. Future investigations, using new open-end next-generation sequencing techniques, should aim to further characterize the role of the oral/periodontal microbiome in the pathobiology of mucositis as well as in systemic infectious complications. Among other factors, the use of prophylactic antibiotics has changed the spectrum of isolated pathogens from the bloodstream as the most common causes of bacteremia in myelosuppressed cancer patients are coagulase-negative staphylococci, viridans group streptococci, and enterococci [9], which may all originate from the periodontium and oral mucosa [31, 37, 46, 115–119]. Particularly, the contribution of *Staphylococcus epidermidis* originating from the oral cavity should be studied.

An intriguing hypothesis that needs further exploration is the concept that inflammatory complications induced by cytotoxic cancer therapies may have a common pathobiological background and share genetically determined risk factors [64]. Inflammation, particularly an imbalance between upregulated pro-inflammatory cytokine pathways and suppressed anti-inflammatory cytokines, may be a major common driver of these complications [120]. In addition, macrophages have been identified to play a critical role in the potential bidirectional links between periodontal and systemic inflammatory diseases [121]. It has been proposed that the presence of inflammation (anywhere in the body) primes for a dysregulated and exaggerated inflammatory response following a subsequent inflammatory stimulus [122]. This “two-hit model” has been hypothesized to underpin a potential association between periodontitis and OM [123]. Moreover, it has been suggested that patients with periodontitis are more prone to developing fever and septic shock syndrome, since endotoxins derived from periodontal pockets may lead to precipitating conditions [124]. These intriguing findings and hypotheses deserve further exploration.

An important clinical question concerns the management of potential oral foci of FN. There is a consensus that patients planned to be treated with high-dose CT with or without HSCT, or those who will be treated with curative (chemo)radiation for head and neck cancers should be referred to a dental professional for a comprehensive oral and periodontal pre-treatment evaluation with appropriate treatment. In patients with head and neck cancers, rigorous elimination of potential foci of infection (i.e., in most cases by extraction) is mandatory in patients with at-risk conditions in the radiation volume [125], whereas in other patient categories, protocols may be less rigid. There is substantial evidence suggesting that intensive oral care performed before and during cancer treatment results in a reduction of infectious complications [24, 53, 66, 68, 126–128]. Elad and coworkers [129] developed a predictive model suggesting that no dental treatment increased the probability of dying due to a dental/periodontal infection in HSCT recipients. In selected cases, treatment of periodontal infections by debridement and reducing the oral bacterial load, and endodontic treatments may be preferred over extractions. In addition, approaches aimed at resolving periodontal inflammation hold significant promise [130]. These interventions may also positively affect OM. When FN (likely) originating from the oral cavity develops during CT treatment, antimicrobial therapy should be preferably based on microbiological evaluation of swabs and/or oral rinsing samples.

In conclusion, current recommendations for the prevention of complications include dental evaluation and work up of high-risk patients as an integrated part of the patient workup and management. Nevertheless, large longitudinal studies are needed to obtain more insight in the relative contribution of oral pathologies to FN and infectious complications. Particularly, in patients treated with myelosuppressive CT for solid tumors information is scarce. In addition, more information is needed on the impact of myelosuppressive CT and other medications (i.e., antibiotics) on the oral microbiome and systemic translocation of oral microorganisms. Lastly, studies should be directed to obtain a better insight in individual risk factors of patients and to develop optimal personalized oral care strategies.
